# An evaluation of the three measurable cardinal objectives of the National Youth Service Corps programme: a survey dataset

**DOI:** 10.12688/f1000research.122328.1

**Published:** 2022-06-14

**Authors:** Valentine J. Owan, Emanuel E. Emanghe, Samuel M. Akpan, David A. Alawa, Victor O. Ebuara, Victor A. Abanyam, Mercy V. Owan, Fidelis A. Unimna, Ikutal Ajigo

**Affiliations:** 1 Department of Educational Foundations, University of Calabar, Calabar, Cross River State, 540271, Nigeria; 2 Ultimate Research Network (URN), Calabar, Cross River State, 540271, Nigeria; 3 Department of Educational Management, University of Calabar, Calabar, Cross River State, 540271, Nigeria; 4 Department of Vocational Education, University of Calabar, Calabar, Cross River State, 540271, Nigeria; 5 Department of Social Science Education, University of Calabar, Calabar, Cross River State, 540271, Nigeria

**Keywords:** Community service, graduates, national service, orientation course, primary assignment

## Abstract

**Background: **The National Youth Service Corps programme is, among other targets, aimed at promoting national inclusiveness and tolerance in a culturally heterogeneous society. Despite the importance of this programme, little has been done to evaluate its degree of success. Where evaluations are done, they are never made public. There is a need for the NYSC programme, just like all other public programmes, to be evaluated for transparency, accountability and decision-making. From an evaluation of the three measurable objectives of the NYSC programme, this dataset bridges this gap **.**

**Methods: **This dataset was collected from Nigerian graduates that completed their national service between 2012 and 2021. The data was collected through an electronic survey posted to various online platforms hosting National Youth Service Corps (NYSC) members of the various sets and batches. The data collection aimed to evaluate the three cardinal objectives of the programme. After three years of data collection (from 2019 to 2021), responses were obtained from 19,278 participants that met the eligibility criteria. The data is an Excel (.xlsx) document with 19,278 cases and 95 variables. Descriptive statistics such as frequency counts and simple percentages were used to summarise the data. However, charts are further used to illustrate the demographic attributes of the respondents. The dataset is broad and covers all the 36 states in Nigeria plus the Federal Capital Territory.

**Results: **The data set has many reuse potentials because it contains information on camp activities (such as parades, military drills, redeployment, quality of food, and facilities, among others), primary assignments and community service engagements of corps members.

**Conclusions**: The data can offer a complete evaluation of how the (NYSC) has attained three of its four cardinal objectives. A series of relationships can further be determined and tested using inferential statistics among the variables included in the dataset.

## Introduction

The National Youth Service Corps (NYSC) was established in 1973 by the Nigerian government under its military rule to include graduates in the construction of Nigeria and the country’s growth. ^1^^,^^2^ The aim of establishing the programme was to foster oneness and selfless service to the Nigerian community. ^3^ After graduating from university or a polytechnic in Nigeria, students are expected to participate in the National Youth Service Corps programme for one year. ^4^ Graduates who are over the age of 30 at the time of graduation will get a Certificate of Exemption, which is the equivalent of the NYSC Discharge Certificate, and will not be forced to do the necessary one-year service. A graduate cannot opt out of NYSC on their own unless they are disabled, have served in the military or paramilitary for more than a year, or are over 30 years old. Part-time graduates get an exemption since they are ineligible for military service.

There are four cardinal areas of the programme – orientation course, primary assignment, community development service and winding-up/passing-out activities. The orientation course includes parade/paramilitary training, physical training, Man ‘O’ War activities, sports/games, language study, kitchen/cooking activities, sanitation and social activities. The orientation course lasts for three weeks (21 days), after which corps members are posted to the various places of primary assignment. Aside from the 21 days meant for camp activities, most of the one-year mandatory service is dedicated to primary assignment and community development service. The winding-up activities enable corps members (who have successfully completed national service) to exit the programme.

**Figure 1.**
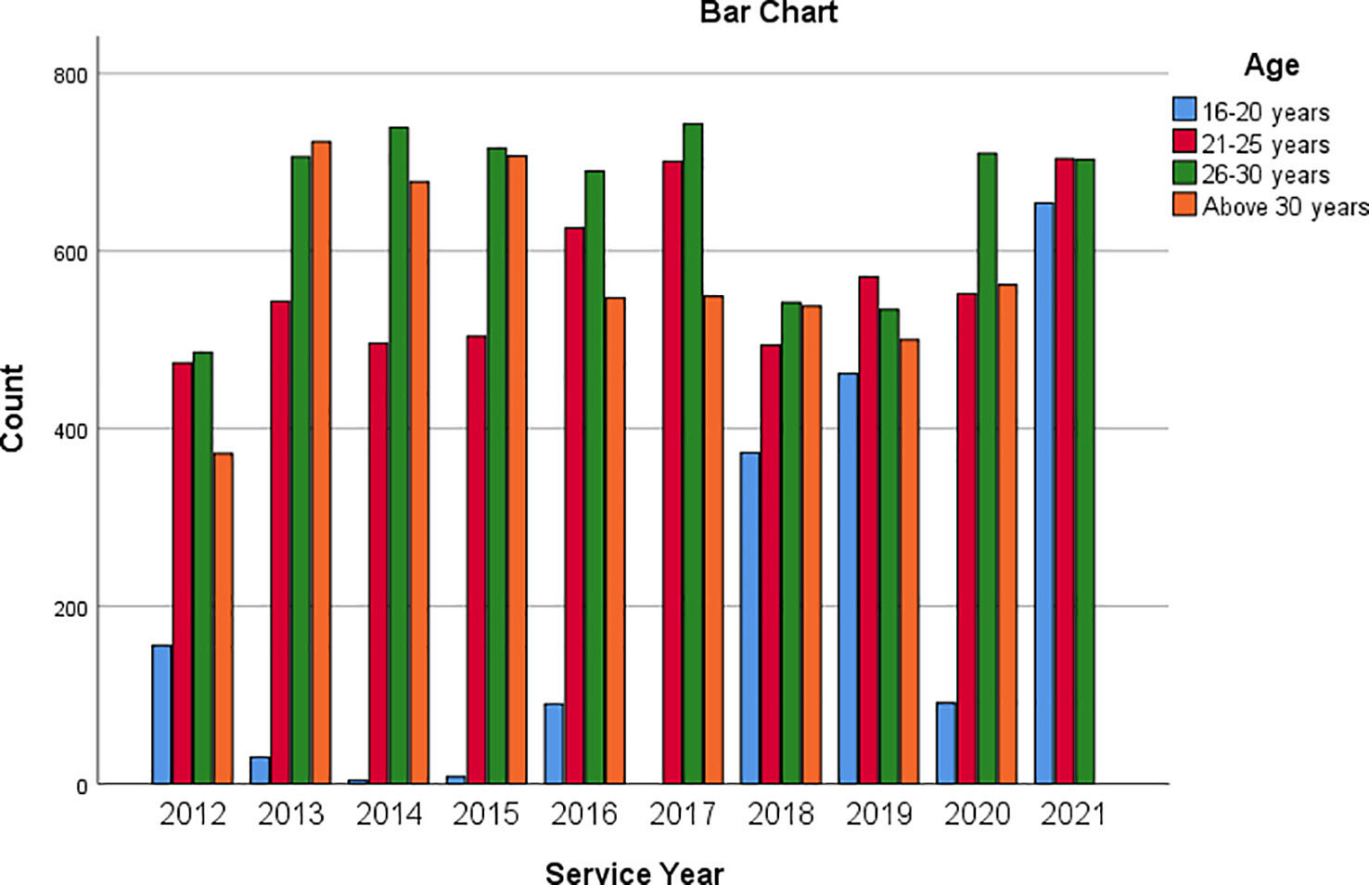
Bar chart showing respondents’ age based on service year.

**Figure 2.**
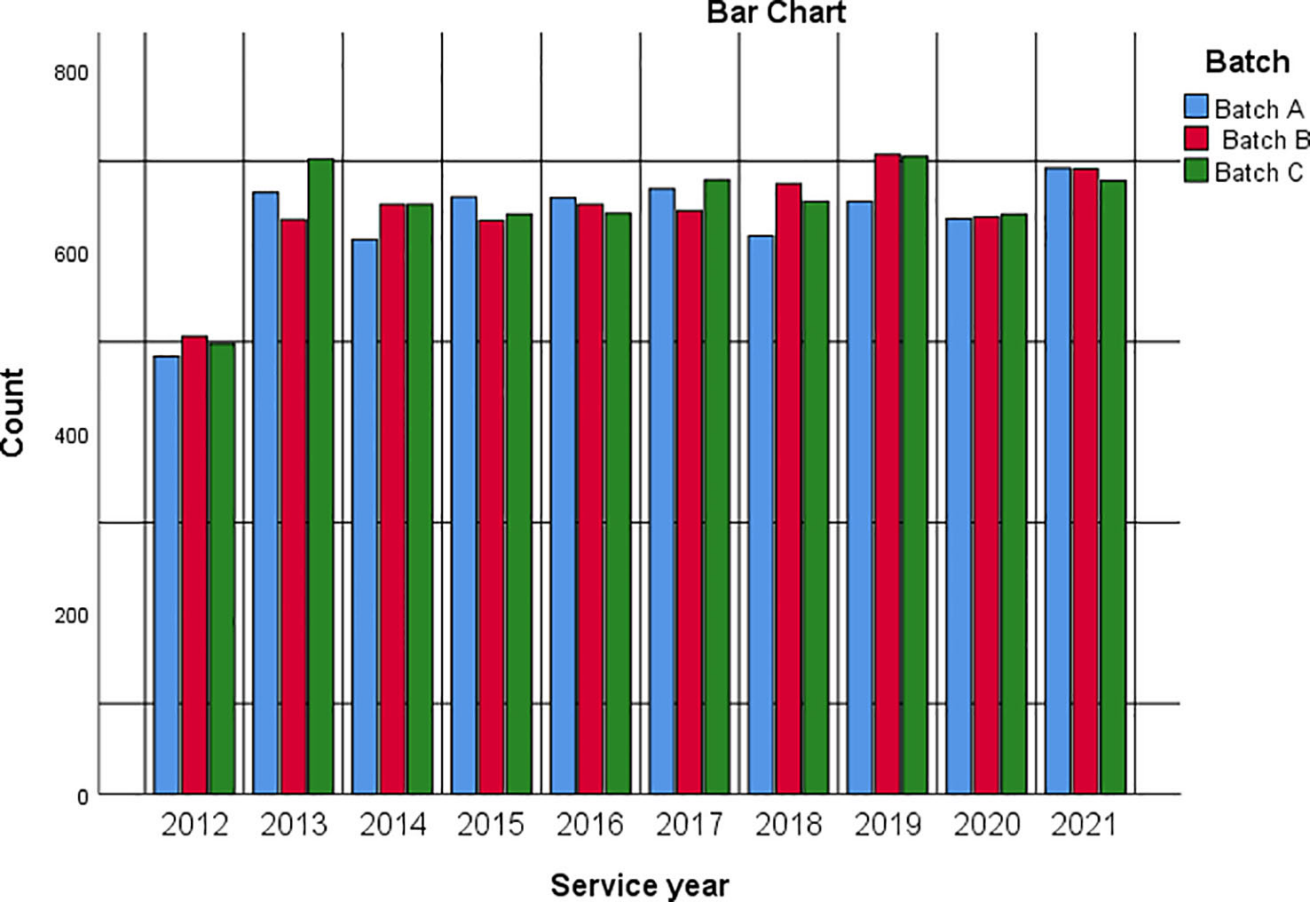
Bar chart showing respondents’ service year based on service batch.

**Figure 3.**
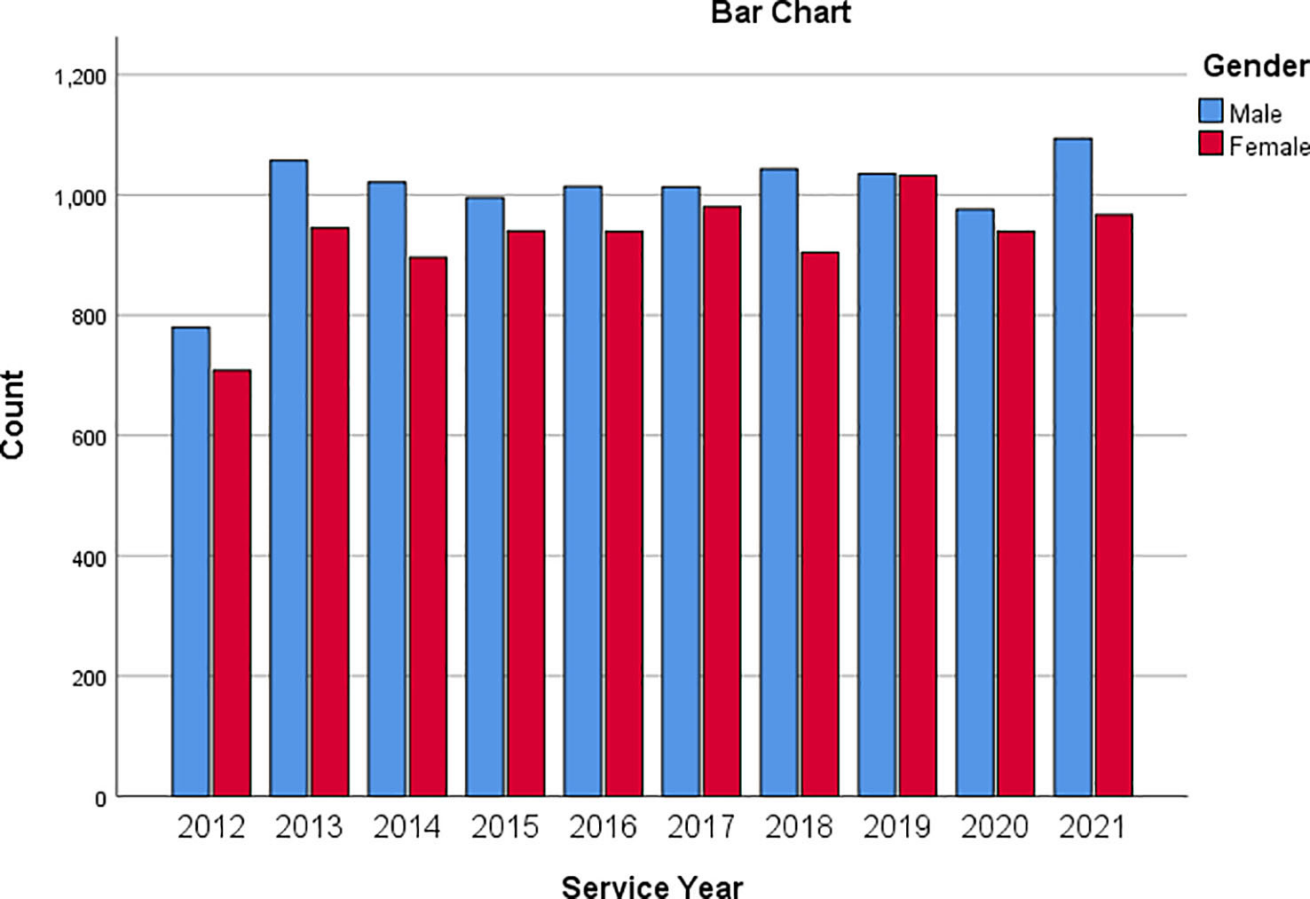
Bar chart showing respondents’ service year based on gender.

Despite the importance of NYSC in promoting national integration and peaceful coexistence, the programme is rarely evaluated, and where it is done, results are not communicated to the public. Thus, it becomes difficult for stakeholders to determine if the programme is successful and the degree of such success. Bridging this gap, we collected this data from a survey evaluating the three important cardinal areas of the NYSC – orientation course, primary assignment and community development service. The project contains two files – excel document (.xlsx) and comma-separated values (.csv) (see *Underlying data*). ^5^ There are 19,278 cases and 95 variables. The first seven columns of the .xlsx file contain data about the demographic variables of the respondents. Table 1 presents the demographic characteristics of respondents such as age, gender, marital status, educational qualification, service year and batch. Respondents provided the demographic variables of their status when filling out the survey and not during their service year. Table 2 is a crosstabulation of respondents’ state of deployment and service year. The crosstabulation is an easy visualisation of the number of corps members that served in the 37 NYSC camps in Nigeria each year from 2012 to 2021. Figure 1 is a multiple bar chart showing the age of respondents across each of the service years covered by the data. Similarly, Figure 2 is a bar chart summarising respondents’ service year and batch intersection. Figure 3 is a bar chart showing the service based on the gender of respondents. This way, one can visualise the number of male and female respondents based on their service year.

**Table 1.** Demographic characteristics of the respondents.

Variable	Levels	Frequency (F)	%
Age	16-20 years	1868	9.7
	21-25 years	5665	29.4
	26-30 years	6569	34.1
	Above 31 years	5176	26.8
	Total	19278	100
Gender	Male	10028	52
	Female	9250	48
	Total	19278	100
Marital status	Married	1600	8.3
	Single	17678	91.7
	Total	19278	100
Educational qualification	Higher National Diploma	3244	16.8
	Bachelor’s Degree	14226	73.8
	Master’s	1718	8.9
	Doctorate	90	0.5
	Total	19278	100
Service year	2012	1488	7.7
	2013	2002	10.4
	2014	1917	9.9
	2015	1935	10
	2016	1953	10.1
	2017	1993	10.3
	2018	1947	10.1
	2019	2067	10.7
	2020	1915	9.9
	2021	2061	10.7
	Total	19278	100
Batch	Batch A	6350	32.9
	Batch B	6435	33.4
	Batch C	6493	33.7
	Total	19278	100

**Table 2.** Respondents’ state of deployment by service.

State of deployment		Service year										
		2012	2013	2014	2015	2016	2017	2018	2019	2020	2021	Total
Abia	N	49	81	93	68	91	79	71	102	62	59	755
	%	3.3	4.0	4.9	3.5	4.7	4.0	3.6	4.9	3.2	2.9	3.9
Adamawa	N	0	13	17	20	11	14	16	11	12	13	127
	%	0.0	0.6	0.9	1.0	0.6	0.7	0.8	0.5	0.6	0.6	0.7
Akwa Ibom	N	0	103	103	97	102	105	105	87	91	107	900
	%	0.0	5.1	5.4	5.0	5.2	5.3	5.4	4.2	4.8	5.2	4.7
Anambra	N	63	117	95	94	104	99	96	145	108	113	1034
	%	4.2	5.8	5.0	4.9	5.3	5.0	4.9	7.0	5.6	5.5	5.4
Bauchi	N	0	52	47	66	54	51	48	44	56	55	473
	%	0.0	2.6	2.5	3.4	2.8	2.6	2.5	2.1	2.9	2.7	2.5
Bayelsa	N	90	78	83	95	94	102	85	96	79	98	900
	%	6.0	3.9	4.3	4.9	4.8	5.1	4.4	4.6	4.1	4.8	4.7
Benue	N	40	35	32	36	34	34	34	40	38	38	361
	%	2.7	1.7	1.7	1.9	1.7	1.7	1.7	1.9	2.0	1.8	1.9
Borno	N	11	8	5	9	9	16	8	14	7	13	100
	%	0.7	0.4	0.3	0.5	0.5	0.8	0.4	0.7	0.4	0.6	0.5
Cross River	N	121	120	137	112	115	112	122	119	106	136	1200
	%	8.1	6.0	7.1	5.8	5.9	5.6	6.3	5.8	5.5	6.6	6.2
Delta	N	54	61	64	55	70	56	62	56	54	68	600
	%	3.6	3.0	3.3	2.8	3.6	2.8	3.2	2.7	2.8	3.3	3.1
Ebonyi	N	34	26	32	23	31	30	30	26	40	28	300
	%	2.3	1.3	1.7	1.2	1.6	1.5	1.5	1.3	2.1	1.4	1.6
Edo	N	42	44	43	51	39	58	41	50	31	46	445
	%	2.8	2.2	2.2	2.6	2.0	2.9	2.1	2.4	1.6	2.2	2.3
Ekiti	N	27	37	30	23	29	26	35	36	23	34	300
	%	1.8	1.8	1.6	1.2	1.5	1.3	1.8	1.7	1.2	1.6	1.6
Enugu	N	89	93	86	89	97	87	105	74	91	89	900
	%	6.0	4.6	4.5	4.6	5.0	4.4	5.4	3.6	4.8	4.3	4.7
FCT	N	60	71	70	73	65	62	81	85	78	70	715
	%	4.0	3.5	3.7	3.8	3.3	3.1	4.2	4.1	4.1	3.4	3.7
Gombe	N	11	28	24	37	32	34	35	37	38	24	300
	%	0.7	1.4	1.3	1.9	1.6	1.7	1.8	1.8	2.0	1.2	1.6
Imo	N	14	34	22	26	30	33	30	42	39	30	300
	%	0.9	1.7	1.1	1.3	1.5	1.7	1.5	2.0	2.0	1.5	1.6
Jigawa	N	76	98	76	76	93	104	96	78	101	102	900
	%	5.1	4.9	4.0	3.9	4.8	5.2	4.9	3.8	5.3	4.9	4.7
Kaduna	N	41	62	68	63	58	58	76	85	67	59	637
	%	2.8	3.1	3.5	3.3	3.0	2.9	3.9	4.1	3.5	2.9	3.3
Kano	N	24	39	28	35	33	32	24	24	28	33	300
	%	1.6	1.9	1.5	1.8	1.7	1.6	1.2	1.2	1.5	1.6	1.6
Katsina	N	15	16	15	18	20	23	24	10	11	14	166
	%	1.0	0.8	0.8	0.9	1.0	1.2	1.2	0.5	0.6	0.7	0.9
Kebbi	N	22	23	24	19	18	26	22	27	22	36	239
	%	1.5	1.1	1.3	1.0	0.9	1.3	1.1	1.3	1.1	1.7	1.2
Kogi	N	23	29	34	26	38	34	24	35	31	26	300
	%	1.5	1.4	1.8	1.3	1.9	1.7	1.2	1.7	1.6	1.3	1.6
Kwara	N	26	50	39	46	49	48	36	46	38	52	430
	%	1.7	2.5	2.0	2.4	2.5	2.4	1.8	2.2	2.0	2.5	2.2
Lagos	N	52	62	53	58	57	58	53	79	68	60	600
	%	3.5	3.1	2.8	3.0	2.9	2.9	2.7	3.8	3.6	2.9	3.1
Nasarawa	N	104	177	170	177	159	180	155	189	176	207	1694
	%	7.0	8.8	8.9	9.1	8.1	9.0	8.0	9.1	9.2	10.0	8.8
Niger	N	23	28	31	29	29	36	38	28	23	35	300
	%	1.5	1.4	1.6	1.5	1.5	1.8	2.0	1.4	1.2	1.7	1.6
Ogun	N	31	31	32	28	24	37	19	29	32	37	300
	%	2.1	1.5	1.7	1.4	1.2	1.9	1.0	1.4	1.7	1.8	1.6
Ondo	N	27	32	29	29	40	29	32	34	26	22	300
	%	1.8	1.6	1.5	1.5	2.0	1.5	1.6	1.6	1.4	1.1	1.6
Osun	N	27	37	28	28	25	37	24	27	34	33	300
	%	1.8	1.8	1.5	1.4	1.3	1.9	1.2	1.3	1.8	1.6	1.6
Oyo	N	65	58	86	83	85	78	80	74	90	85	784
	%	4.4	2.9	4.5	4.3	4.4	3.9	4.1	3.6	4.7	4.1	4.1
Plateau	N	22	34	32	41	27	26	29	35	26	28	300
	%	1.5	1.7	1.7	2.1	1.4	1.3	1.5	1.7	1.4	1.4	1.6
Rivers	N	68	69	48	62	69	63	53	52	60	56	600
	%	4.6	3.4	2.5	3.2	3.5	3.2	2.7	2.5	3.1	2.7	3.1
Sokoto	N	8	5	3	8	9	7	5	7	8	10	70
	%	0.5	0.2	0.2	0.4	0.5	0.4	0.3	0.3	0.4	0.5	0.4
Taraba	N	22	29	32	29	27	26	35	42	28	30	300
	%	1.5	1.4	1.7	1.5	1.4	1.3	1.8	2.0	1.5	1.5	1.6
Yobe	N	59	54	43	51	45	38	54	48	38	55	485
	%	4.0	2.7	2.2	2.6	2.3	1.9	2.8	2.3	2.0	2.7	2.5
Zamfara	N	48	68	63	55	41	55	64	54	55	60	563
	%	3.2	3.4	3.3	2.8	2.1	2.8	3.3	2.6	2.9	2.9	2.9
Total	N	1488	2002	1917	1935	1953	1993	1947	2067	1915	2061	19278
	%	100	100	100	100	100	100	100	100	100	100	100

Columns 8 to 40 of the data provide corps members’ experience-based data on different aspects of orientation course exercises. Tables 3 and 4 summarise responses and percentages to different orientation course exercises such as nature of camp officials, man ‘o’ war drills, eating frequency, quality of food, and availability of camp facilities. Columns 41 to 53 of the data contain data about the NYSC kits given to corps members in camp. Tables 5 and 6 summarise data on the quantity and quality of kits given to corps members. Column 54 contains data on the allowances provided to corps members while in camp. Column 55 contains dichotomously scored data from a question asking whether respondents’ thoughts the 21 days (3 weeks) of the camp experience were adequate or otherwise. Columns 56 to 67 contained polytomous data scored from responses to four-point Likert scale items on the Skill Acquisition and Entrepreneurship Development (SAED) initiative of NYSC. Table 7 presents a summary of the responses to all the SAED items.

**Table 3.** Likert-scale items on respondents' orientation course experiences.

SN	Items	SA [%]	A [%]	D [%]	SD [%]	Total [%]
1	Camp officials made efforts to ensure that everyone took an active part in activities.	9491 [49.2]	9487 [49.2]	159 [0.8]	141 [0.7]	19278 [100]
2	Proper guidance was given to me regarding using Man 'o' war gadgets in my camp.	7995 [41.5]	8114 [42.1]	1568 [8.1]	1601 [8.3]	19278 [100]
3	Camp officials usually ensured that the camp environment was neat and well kept.	9197 [47.7]	9098 [47.2]	485 [2.5]	498 [2.6]	19278 [100]
4	I was provided with a sleeping mattress while in camp.	9185 [47.6]	9110 [47.3]	481 [2.5]	502 [2.6]	19278 [100]
5	The quality of food served in my camp was very delicious.	4456 [23.1]	4410 [22.9]	5203 [27.0]	5209 [27.0]	19278 [100]
6	Social gatherings/events were organised in my camp for corps members.	8901 [46.2]	9007 [46.7]	713 [3.7]	657 [3.4]	19278 [100]
7	I was taught a new language of the immediate environment while in camp.	7122 [36.9]	7179 [37.2]	2461 [12.8]	2516 [13.1]	19278 [100]
8	There were no issues of electricity challenges in my camp.	5098 [26.4]	5183 [26.9]	4503 [23.4]	4494 [23.3]	19278 [100]
9	Corps members were not allowed to dress as they liked in my camp.	7575 [39.3]	7548 [39.2]	2082 [10.8]	2073 [10.8]	19278 [100]
10	The mami market of my camp was superb in meeting the diverse needs of corps members.	7643 [39.6]	7786 [40.4]	1910 [9.9]	1939 [10.1]	19278 [100]
11	Corp members were allowed to take part in cooking activities in my camp.	7839 [40.7]	7803 [40.5]	1784 [9.3]	1852 [9.6]	19278 [100]
12	It was possible to stay in my camp without doing anything.	4272 [22.2]	4258 [22.1]	5346 [27.7]	5402 [28.0]	19278 [100]
13	Some members of my platoon did not participate in any camp activity.	6592 [34.2]	6697 [34.7]	2993 [15.5]	2996 [15.5]	19278 [100]
14	Some Man' O' war gadgets in my camp were not in good shape.	3946 [20.5]	4027 [20.9]	5668 [29.4]	5637 [29.2]	19278 [100]
15	Many foam/beds in my camp were in poor condition.	6290 [32.6]	6229 [32.3]	3371 [17.5]	3388 [17.6]	19278 [100]
16	The number of functional boreholes/tabs were not adequate for the number of corps members in camp.	6494 [33.7]	6751 [35.0]	2918 [15.1]	3115 [16.2]	19278 [100]
17	Meals were not served consistently, at least three times per day in my camp.	4209 [21.8]	4248 [22.0]	5495 [28.5]	5326 [27.6]	19278 [100]
18	There were no sports activities throughout my stay at camp.	1441 [7.5]	1430 [7.4]	8090 [42.0]	8317 [43.1]	19278 [100]
19	I did not obey any difficult camp instructions.	3549 [18.4]	3434 [17.8]	6180 [32.1]	6115 [31.7]	19278 [100]
20	The medical centre of my camp is nothing to write home about.	3121 [16.2]	3178 [16.5]	6466 [33.5]	6513 [33.8]	19278 [100]
21	The military men on duty in my camp were highly unfriendly.	2680 [13.9]	2679 [13.9]	6833 [35.4]	7086 [36.8]	19278 [100]
22	The discipline approaches of camp officers were very harsh on corps members.	2636 [13.7]	2543 [13.2]	7031 [36.5]	7068 [36.7]	19278 [100]
23	Prices of goods and services in my camp were very high.	5906 [30.6]	5960 [30.9]	3625 [18.8]	3787 [19.6]	19278 [100]
24	Sanitation activities in my camp were not carried out daily.	3348 [17.4]	3252 [16.9]	6388 33.1]	6290 [32.6]	19278 [100]

Note: SA = Strongly agree; A = Agree; D = Disagree; SD = Strongly disagree.

**Table 4.** Other questions regarding camp experience by corps members.

Question	Response	N	%
Was there any form of parade or paramilitary training in your camp?	Yes	18892	98
	No	386	2
	Total	19278	100
Was there immediate treatment for sick corps members admitted to the camp clinic?	Yes	14103	73.2
	To some extent	5095	26.4
	No	80	0.4
	Total	19278	100
How will you rate the adequacy of the treatment rendered by the camp clinic to sick corps members?	1 (Very inadequate)	473	2.5
	2 (Inadequate)	4100	21.3
	3 (Adequate)	10377	53.8
	4 (Very adequate)	4328	22.5
	Total	19278	100
Did you participate in parade activities	Yes	15640	81.1
	Sometimes	1373	7.1
	No	2265	11.7
	Total	19278	100
How often did you eat camp food	Always	7506	38.9
	Sometimes	9672	50.2
	Never	2100	10.9
	Total	19278	100
If you never ate camp food at all, what were your reasons?	I was not always comfortable going to collect food	22	0.1
	I was not in camp due to exit permission that was granted	16	0.1
	I was with cash to get whatever I wanted	35	0.2
	Most meals were not deliciously prepared	600	3.1
	Poor quality of food served	1196	6.2
	The food served most times are not my choice	341	1.8
	Total	17068	88.5
If you ate camp food sometimes, what were your reasons for avoiding camp food sometimes?	I avoided only beans while at camp	600	3.1
	I avoided some food due to allergies	111	0.6
	I disliked the toilet condition and avoided what could trigger frequent toilet	67	0.3
	I had a better option	609	3.2
	I had a running stomach after eating camp food	300	1.6
	I hate camp food sometimes because of the officials and their problems	300	1.6
	I limited what I ate to majorly carbohydrates	162	0.8
	I only ate camp food because I had no money	367	1.9
	I only ate nicely prepared meals while I avoided poorly prepared ones	873	4.5
	I only ate rice	727	3.8
	I only took camp tea that enriched by myself with additional milk	38	0.2
	I really enjoyed camp food	300	1.6
	I was not always satisfied with the quantity of food served	34	0.2
	I was not impressed with the food prepared most times	900	4.7
	Most meals were poorly prepared	1837	9.5
	No appetite for some food due to their uninviting physical appearance	329	1.7
	Some food did not meet my taste	300	1.6
	Sometimes I get carried away by social activities while food is served	300	1.6
	Sometimes they did not cook what I wanted	600	3.1
	The distance from my hostel to where the food was served was too far	184	1
	The food contained too many spices	262	1.4
	The food was unhealthy	600	3.1
	Watery and tasteless meals were often prepared	106	0.5
	Total	9372	48.6

**Table 5.** Participants’ responses on the quantity (Qty) of kits given to them during NYSC service.

Kits	Qty given	Frequency	%	Qty required	
NYSC Cap	1 piece	18892	98	1 piece	
	2 pieces	386	2		
	Total	19278	100		
Khaki Trouser	1 piece	18378	95.3	1 piece	
	2 pieces	900	4.7		
	Total	19278	100		
NYSC Belt	1 piece	19192	99.6	1 piece	
	2 pieces	86	0.4		
	Total	19278	100		
Jungle boots	1 piece	18329	95.1	1 piece	
	2 pieces	949	4.9		
	Total	19278	100		
NYSC Crested vest	1 piece	18116	94	1 piece	
	2 pieces	1162	6		
	Total	19278	100		
White Canvas	1 piece	17991	93.3	1 piece	
	2 pieces	1287	6.7		
	Total	19278	100		
P.E. Shorts	1 piece	3428	17.8	2 pieces	
	2 pieces	15850	82.2		
	Total	19278	100		
Plain vest	1 piece	5276	27.4	2 pieces	
	2 pieces	14002	72.6		
	Total	19278	100		
NYSC Socks	1 piece	7453	38.7	2 pieces	
	2 pieces	11825	61.3		
	Total	19278	100		
Khaki Jacket	1 piece	18378	95.3	1 piece	
	2 pieces	900	4.7		
	Total	19278	100		
		**Yes**	**Somehow**	**No**	**Total**
Did NYSC provide you with kits according to your specification on the green card		2959	3262	13057	19278
		15.3	16.9	67.7	100
Were your kits durable? (i.e., did they serve you throughout the camp and beyond?)		10674		8604	19278
		55.4		44.6	100

**Table 6.** Respondents given different combinations of under/oversized NYSC kits.

SN	Kits given under/oversized	N	%
1	Crested vest, Jungle boots, Canvass, P.E. shorts, Plain vest	300	1.6
2	Crested vest, Khaki (Trouser), Jungle boots	300	1.6
3	Crested vest, Khaki (Trouser), Jungle boots, Khaki (Jacket)	600	3.1
4	Crested vest, Khaki (Trouser), Jungle boots, Socks, Plain vest, Khaki (Jacket)	300	1.6
5	Crested vest, Khaki (Trouser), Jungle boots, Canvass, Khaki (Jacket)	686	3.6
6	Crested vest, Khaki (Trouser), Jungle boots, Canvass, P.E. shorts, Khaki (Jacket)	600	3.1
7	Crested vest, Khaki (Trouser), Jungle boots, Canvass, P.E. shorts, Socks, Plain vest, Khaki (Jacket)	86	0.4
8	Crested vest, Khaki (Trouser), Jungle boots, Canvass, P.E. shorts, Socks, Plain vest, Belt, Khaki (Jacket)	600	3.1
9	Crested vest, Khaki (Trouser), Jungle boots, Canvass, P.E. shorts, Plain vest, Khaki (Jacket)	87	0.5
10	Crested vest, Khaki (Trouser), Jungle boots, Canvass, Plain vest, Khaki (Jacket)	300	1.6
11	Crested vest, Khaki (Trouser), Jungle boots, P.E. shorts, Khaki (Jacket)	300	1.6
12	Crested vest, Khaki (Trouser), Jungle boots, P.E. shorts, Plain vest, Khaki (Jacket)	300	1.6
13	Crested vest, Khaki (Trouser), Jungle boots, Plain vest, Khaki (Jacket)	300	1.6
14	Crested vest, Khaki (Trouser), Khaki (Jacket)	300	1.6
15	Crested vest, Khaki (Trouser), Canvass, Khaki (Jacket)	83	0.4
16	Cap, Crested vest, Khaki (Trouser), Jungle boots, Canvass, P.E. shorts, Socks, Plain vest, Khaki (Jacket)	300	1.6
17	Cap, Crested vest, Khaki (Trouser), Jungle boots, Canvass, P.E. shorts, Socks, Plain vest, Belt, Khaki (Jacket)	2317	12
18	Cap, Crested vest, Khaki (Trouser), Jungle boots, Canvass, Plain vest, Khaki (Jacket)	300	1.6
19	Cap, Crested vest, Khaki (Trouser), Canvass, Belt	300	1.6
20	Cap, Crested vest, Khaki (Trouser), Canvass, P.E. shorts, Socks, Plain vest, Belt, Khaki (Jacket)	300	1.6
21	Cap, Crested vest, Khaki (Trouser), Canvass, Plain vest, Khaki (Jacket)	87	0.5
22	Cap, Crested vest, Khaki (Trouser), Plain vest, Khaki (Jacket)	300	1.6
23	Cap, Khaki (Trouser), Jungle boots, Canvass, Khaki (Jacket)	300	1.6
24	Cap, Khaki (Trouser), Jungle boots, Canvass, Socks, Khaki (Jacket)	300	1.6
25	Cap, Khaki (Trouser), Jungle boots, Canvass, Plain vest, Khaki (Jacket)	87	0.5
26	Cap, Khaki (Trouser), Jungle boots, P.E. shorts, Plain vest, Khaki (Jacket)	300	1.6
27	Cap, Khaki (Trouser), Canvass, Plain vest, Khaki (Jacket)	82	0.4
28	Jungle boots	900	4.7
29	Jungle boots, Canvass, Socks, Plain vest	83	0.4
30	Jungle boots, Canvass, P.E. shorts, Socks, Plain vest	300	1.6
31	Khaki (Trouser)	86	0.4
32	Khaki (Trouser), Jungle boots, Khaki (Jacket)	900	4.7
33	Khaki (Trouser), Jungle boots, Canvass, Khaki (Jacket)	1761	9.1
34	Khaki (Trouser), Jungle boots, Canvass, P.E. shorts, Khaki (Jacket)	900	4.7
35	Khaki (Trouser), Jungle boots, Canvass, P.E. shorts, Belt, Khaki (Jacket)	300	1.6
36	Khaki (Trouser), Jungle boots, Canvass, P.E. shorts, Plain vest, Khaki (Jacket)	166	0.9
37	Khaki (Trouser), Jungle boots, Canvass, Plain vest, Khaki (Jacket)	86	0.4
38	Khaki (Trouser), Jungle boots, P.E. shorts, Khaki (Jacket)	300	1.6
39	Khaki (Trouser), Jungle boots, Plain vest	82	0.4
40	Khaki (Trouser), Jungle boots, Plain vest, Khaki (Jacket)	300	1.6
41	Khaki (Trouser), Khaki (Jacket)	386	2
42	Khaki (Trouser), Canvass	384	2
43	Khaki (Trouser), Canvass, Khaki (Jacket)	600	3.1
44	Khaki (Trouser), Canvass, P.E. shorts, Khaki (Jacket)	300	1.6
45	Khaki (Trouser), Canvass, P.E. shorts, Socks, Plain vest, Khaki (Jacket)	300	1.6
46	Khaki (Trouser), Canvass, P.E. shorts, Plain vest, Khaki (Jacket)	300	1.6
47	Khaki (Trouser), Canvass, Plain vest	167	0.9
48	Khaki (Trouser), P.E. shorts, Socks, Khaki (Jacket)	262	1.4
49	P.E. shorts, Socks, Plain vest	300	1.6
	Total	19278	100

**Table 7.** Respondents’ experiences on skill acquisition and entrepreneurship development (SAED) initiative of NYSC.

SN	Items	SA [%]	A [%]	D [%]	SD [%]	Total [%]
1	There were adequate seats in the multi-purpose hall of my camp for SAED lectures	6484 [33.6]	6414 [33.3]	3217 [16.7]	3163 [16.4]	19278 [100]
2	NYSC provided sources of loans for corps members to assess funds to start their businesses	4947 [25.7]	4912 [25.5]	4702 [24.4]	4717 [24.5]	19278 [100]
3	There were no SAED lectures in my camp	7951 [41.2]	7680 [39.8]	1813 [9.4]	1834 [9.5]	19278 [100]
4	Many corps members in my camp often sit outside the hall while SAED lectures are going on.	2557 [13.3]	2485 [12.9]	7134 [37.0]	7102 [36.8]	19278 [100]
5	The SAED lectures in my camp were more of theory than practical.	3549 [18.4]	3642 [18.9]	6088 [31.6]	5999 [31.1]	19278 [100]
6	I did not acquire any practical skills through the SAED lectures.	3172 [16.5]	9164 [47.5]	3556 [18.4]	3386 [17.6]	19278 [100]
7	My SAED lecturer was not competent in presenting clear lessons.	6656 [34.5]	6625 [34.4]	3076 [16.0]	2921 [15.2]	19278 [100]
8	There were inadequate facilities for every corps member in my SAED venture to conduct practicals with	3255 [16.9]	3253 [16.9]	6285 [32.6]	6485 [33.6]	19278 [100]
9	The duration of SAED training was too short for me to acquire the requisite skills for my venture	2531 [13.1]	2136 [11.1]	6645 [34.5]	7966 [41.3]	19278 [100]
10	I was charged additional fees to advance my skills in my chosen trade after camp.	3452 [17.9]	3572 [18.5]	6045 [31.4]	6209 [32.2]	19278 [100]
				**Yes**	**No**	**Total**
11	Did the SAED lectures enable you to acquire a new set of skills?			12713 [65.9]	6565 [34.1]	19278 [100]
12	Are you currently using the skill you acquired during service to support your livelihood?			3987 [20.7]	15291 [79.3]	19278 [100]

Note: SA = Strongly agree; A = Agree; D = Disagree; SD = Strongly disagree.

Column 68 of the raw dataset presents reasons for a follow-up question (see serial number 13 in Table 7) explaining why some respondents are not using their skills acquired through SAED during service. These reasons are provided in Table 8 as a combination and the frequency of respondents with such combinations of reasons. Columns 69 to 85 contain data scored from four-point Likert scale items on respondents’ experiences with their Place of Primary Assignment (PPA), which is the second cardinal objective of the NYSC. Table 9 summarises the frequency-based data on respondents’ attitudes and experiences with their PPA.

**Table 8.** Combinations of reasons why corps members are not using the skills acquired from NYSC.

SN	Reasons	N	%
1	I cannot make use of my skills because I feel it is a waste of time	300	1.6
2	I have a better job/business prospect I am considering than the skills I acquired	649	3.4
3	I intend to start in the future	1800	9.3
4	I intend to start in the future; the skills I acquired were forgotten due to the short duration of time given to practicals.	300	1.6
5	The skills I acquired were not adequate to compete with others in the same line of business	2913	15.1
6	The skills I acquired were not adequate to compete with others in the same line of business; I have a better job/business prospect I am considering than the skills I acquired.	300	1.6
7	The skills I acquired were not adequate to compete with others in the same line of business; I intend to start in the future; I want to further my education first before using my skills.	600	3.1
8	The skills I acquired were not adequate to compete with others in the same line of business; there is no capital to start a business requiring my skills.	1280	6.6
9	The skills I acquired were not adequate to compete with others in the same line of business; there is no capital to start a business requiring my skills; I cannot make use of my skills because I feel it is a waste of time.	83	0.4
10	The skills I acquired were not adequate to compete with others in the same line of business; there is no capital to start a business requiring my skills; I have a better job/business prospect I am considering than the skills I acquired.	982	5.1
11	The skills I acquired were not adequate to compete with others in the same line of business; There is no capital to start a business requiring my skills; I have a better job/business prospect I am considering than the skills I acquired; I cannot make use of my skills because I feel it is a waste of time.	300	1.6
12	The skills I acquired were not adequate to compete with others in the same line of business; there is no capital to start a business requiring my skills; I have a better job/business prospect I am considering than the skills I acquired; I intend to start in the future; I cannot make use of my skills because I feel it is a waste of time; I want to further my education first before using my skills	600	3.1
13	The skills I acquired were not adequate to compete with others in the same line of business; there is no capital to start a business requiring my skills; I intend to start in the future.	83	0.4
14	The skills I acquired were not adequate to compete with others in the same line of business; there is no capital to start a business requiring my skills; I intend to start in the future; I want to further my education first before using my skills	300	1.6
15	The skills I acquired were not adequate to compete with others in the same line of business; there is no capital to start a business requiring my skills; I intend to start in the future; I will still have to go for further education on the skill when I want to start-up in the future.	300	1.6
16	The skills I acquired were not adequate to compete with others in the same line of business; there is no capital to start a business requiring my skills; I did not learn the skills to the standard since there was not enough time to learn the skills	300	1.6
17	There is no capital to start a business requiring my skills	3001	15.6
18	There is no capital to start a business requiring my skills; I have a better job/business prospect; I am considering something more than the skills I acquired.	300	1.6
19	There is no capital to start a business requiring my skills; I have a better job/business prospect; I am considering something more than the skills I acquired; I intend to start in the future.	300	1.6
20	There is no capital to start a business requiring my skills; I intend to start in the future.	300	1.6
21	There is no capital to start a business requiring my skills; I intend to start in the future; I want to further my education first before using my skills	300	1.6
	Total	3601	18.7

**Table 9.** Respondents’ experiences in their places of primary assignment (PPA).

SN	Items	SA [%]	A [%]	D [%]	SD [%]	Total [%]
1	I was posted to a PPA outside my area of speciality.	3137 [16.3]	3112 [16.1]	6467 [33.5]	6562 [34.0]	19278 [100]
2	It took me some time to consider my PPA before reporting to avoid mistakes.	2989 [15.5]	2844 [14.8]	6653 [34.5]	6792 [35.2]	19278 [100]
3	I was instantly rejected at my PPA for no apparent reason.	755 [3.9]	745 [3.9]	8810 [45.7]	8968 [46.5]	19278 [100]
4	The workload assigned to me at my PPA was too unbearable.	2886 [15.0]	2766 [14.3]	6845 [35.5]	6781 [35.2]	19278 [100]
5	There was no accommodation in my PPA.	5537 [28.7]	5422 [28.1]	4167 [21.6]	4152 [21.5]	19278 [100]
6	I travelled each time I liked without the approval of the State coordinator.	1214 [6.3]	1268 [6.6]	8471 [43.9]	8325 [43.2]	19278 [100]
7	I was paying PPA officials to give me monthly clearance letters due to my busy schedule.	450 [2.3]	450 [2.3]	9144 [47.4]	9234 [47.9]	19278 [100]
8	I changed my original PPA to another PPA with better welfare	1445 [7.5]	1420 [7.4]	8286 [43.0]	8127 [42.2]	19278 [100]
9	Some corps members in my PPA were rude to the organisation's leadership.	2801 [14.5]	2899 [15.0]	6748 [35.0]	6830 [35.4]	19278 [100]
10	Some corps members in my PPA were persistently late to work.	3629 [18.8]	3471 [18.0]	6076 [31.5]	6102 [31.7]	19278 [100]
11	Distance from PPA prevented many corps members from attending work on time.	3460 [17.9]	3478 [18.0]	6064 [31.5]	6276 [32.6]	19278 [100]
12	I was reported to the LGI/LI by my PPA only on a few occasions.	800 [4.1]	783 [4.1]	8790 [45.6]	8905 [46.2]	19278 [100]
13	I accepted my PPA without any question.	8469 [43.9]	8627 [44.8]	1104 [5.7]	1078 [5.6]	19278 [100]
14	My PPA consistently paid monthly stipends to her corps members.	4625 [24.0]	4548 [23.6]	5124 [26.6]	4981 [25.8]	19278 [100]
15	I was never queried in my PPA throughout my time with the organisation.	7377 [38.3]	7568 [39.3]	2109 [10.9]	2224 [11.5]	19278 [100]
16	I seldom absent myself from duties at my PPA without prior permission from the management …	2121 [11.0]	2073 [10.8]	7589 [39.4]	7495 [38.9]	19278 [100]
17	I was paid all my stipends at my PPA/state government.	6057 [31.4]	5893 [30.6]	3637 [18.9]	3691 [19.1]	19278 [100]

Note: SA = Strongly agree; A = Agree; D = Disagree; SD = Strongly disagree.

Lastly, columns 86 to 95 of the raw dataset contain polytomous data scored from responses to ten four-point Likert scale items on corps members’ Community Development Services (CDS) during their national service. Table 10 summarises the frequency and percentages of responses to CDS items. Note that the data in columns 9 to 34, 56 to 67, 69 to 85, 86 to 95 can be summed or averaged to obtain a continuous data for the application of other descriptive statistical methods (such as a mean, standard deviation etc.) and/or inferential statistics (such as regression, correlation, structural equation modelling etc.) beyond the frequency summary in Tables 3, 7, 9, and 10 respectively.

**Table 10.** Community development service (CDS) experiences of respondents.

SN	Items	SA [%]	A [%]	D [%]	SD [%]	Total [%]
1	I was not allowed to choose my CDS group at the secretariat.	6703 [34.8]	6697 [34.7]	2927 [15.2]	2951 [15.3]	19278 [100]
2	I belonged to more than one CDS group while serving.	753 [3.9]	733 [3.8]	8865 [46.0]	8927 [46.3]	19278 [100]
3	I was not able to carry out a personal CDS while serving.	5544 [28.8]	5841 [30.3]	3909 [20.3]	3984 [20.7]	19278 [100]
4	My personal CDS activities affected my effectiveness in my PPA.	0 [0.0]	0 [0.0]	9641 [50.0]	9637 [50.0]	19278 [100]
5	The CDS supervising officer did not sign my clearance card sometimes.	0 [0.0]	0 [0.0]	9623 [49.9]	9655 [50.1]	19278 [100]
6	I was unable to contribute to my group CDS because I was busy with my PPA work.	0 [0.0]	0 [0.0]	9490 [49.2]	9788 [50.8]	19278 [100]
7	There was poor response from the host community in funding projects initiated by my CDS group.	4044 [21.0]	4096 [21.2]	5529 [28.7]	5609 [29.1]	19278 [100]
8	There was no cooperation in my CDS group towards initiating viable projects.	1425 [7.4]	1410 [7.3]	8107 [42.1]	8336 [43.2]	19278 [100]
9	I did not miss any of my group CDS activities.	7555 [39.2]	7595 [39.4]	2099 [10.9]	2029 [10.5]	19278 [100]
10	There was active collaboration between my CDS group and members of the host community.	8099 [42.0]	7882 [40.9]	1638 [8.5]	1659 [8.6]	19278 [100]

Note: SA = Strongly agree; A = Agree; D = Disagree; SD = Strongly disagree.

## Value of the data

• This data is helpful because every public programme must be evaluated to determine how short- and long-term objectives are met. The NYSC as a public programme needs to be evaluated to determine its strengths and weaknesses for improvement.
• The Federal Government of Nigeria, the Ministry of youth and sports, the national and state coordinators of the NYSC programme, Nigerian graduates, and the Nigerian community can benefit from this data. The data will open the eyes of stakeholders and the public to the programme’s activities and the extent to which they are successful.
• The data has many reuse potentials because it can enable interested researchers to analyse how various activities are implemented in the programme. Researchers can also relate two or more variables to determine the degree of association.
• The data can further quantify how the programme has empowered graduates with vocational skills through its Skill Acquisition and Entrepreneurship Development (SAED) initiative.
• Demographic variations in corps members’ behaviours towards various activities (such as parade, drills, eating frequency, camp food avoidance, redeployment, truancy etc.) can be estimated using the data. Dimension reduction techniques such as principal component analysis or factor analysis can be applied to the items in the questionnaire to understand the internal structure.
• The effectiveness of all the NYSC camps in Nigeria can be determined using this dataset. Besides, further analysis can be performed at the national and specific camp levels for diagnostic, reward or remediation purposes. Furthermore, the questionnaire associated with the data can be used by other scholars for similar projects in the future.

## Methods

### Ethics

Participation in this research was entirely optional. According to the Nigerian Code of Health Research Ethics (NCHRE), survey-based research is free from the ethical review due to the lack of possible dangers. ^10^ During the data collecting process, written informed consent was collected from respondents. Data collected were anonymised and de-identified per the Safe Harbour Principles. All replies were aggregated with all identifiable information deleted to ensure the data’s integrity and privacy. ^11^

In addition, all biodata, including age, education, and experience, were represented as a range of equal intervals. The questionnaire was also constructed in a way that sensitive information such as respondents’ email addresses, phone numbers, and names were not required. All coded data were saved on the lead researcher’s computer with a strong password, antivirus software, and a firewall to prevent unauthorised access. Respondents were notified that the obtained data will be utilised for academic reasons and that aggregated data may be included in the report published in a peer-reviewed journal. Lastly, respondents were informed that the data would be erased using software from a third party upon the survey’s conclusion.

The survey research design was used in collecting the data. An electronic questionnaire was designed using Google form for data collection (see *Extened data*). ^5^ The researchers drafted the items in the questionnaire through their experiences (having all been past members of NYSC). The experiences pooled from all the researchers, with the support derived from a literature review, were instrumental in developing the first draft of the instrument. The instrument’s draft copy (which was initially on paper) was given to a group of 10 members of NYSC in batch B of 2019 to respond and provide feedback. Their feedback and suggestions were incorporated in developing the final draft copy. The instrument was trial tested on 60 batch C corps members in Nasarawa and FCT. The focus group discussion and trial test respondents were excluded from the main study to avoid ‘testwiseness’. ^6^^–^^9^ Their responses to the Likert scale items were subjected to a reliability analysis of internal consistency using the Cronbach alpha approach. Reliability coefficients of .90, .87, .92, and .89 were obtained for the orientation course, SAED, primary assignment, and CDS.

Primary data for the main study was collected by a snowball sampling process. The total sample was 19,278 previous members of NYSC who served between 2012 and 2021. The aim was to consider corps members within the last decade. The electronic data collection procedure was followed since a link to the survey was shared with close contacts (who had completed their service). Thereafter, the link to the survey was posted to WhatsApp, Telegram, and open Facebook groups of previous and active members of NYSC. The data collection process started in March 2019 and ended in December 2021. All 2019, 2020 and 2021 batches responded while in service, whereas those who served in 2018 or earlier responded after service. Respondents were asked to share the link with their colleagues and post it to their State, Local Government and respective community development (CDS) NYSC groups. The snowballing process expanded as new participants invited other newer members. We stopped the data collection process when we were sure no further responses were forthcoming. We ensured that respondents who were not eligible did not participate by restricting the service year to cover 2012 – 2021 batches. The data collected was downloaded from the cloud, cleaned, qualitative data was converted into numerical forms and recoded using MS-Excel 2019. There was no missing data since all items were compulsory except for optional follow-up questions. Descriptive statistics such as frequency counts and charts were used to analyse the data. However, other statistical methods can be used on the raw data for more insights.

## Data availability

### Underlying data

Mendeley: An Evaluation of the Three Measurable Cardinal Areas of the National Youth Service Corps Programme: A Survey Dataset. https://doi.org/10.17632/jn2t9gw3vt.1 ^5^

The project contains the following underlying data:

• An Evaluation of the three measurable cardinal areas of the National Youth Service Corps programme A survey data cleaned.csv
• An Evaluation of the three measurable cardinal areas of the National Youth Service Corps programme A survey data cleaned.xlsx

### Extended data

Mendeley: An Evaluation of the Three Measurable Cardinal Areas of the National Youth Service Corps Programme: A Survey Dataset. https://doi.org/10.17632/jn2t9gw3vt.1 ^5^

This project contains the following extended data:

• Survey on National Youth Service Corps’ (NYSC) Cardinal Programmes (SNYSCCP).pdf

Data are available under the terms of the Creative Commons Attribution 4.0 International license (CC-BY 4.0).
