# Supplementary material for: An evaluation of the three measurable cardinal objectives of the National Youth Service Corps programme: a survey dataset
Source: F1000Res. 2022 Aug 22;11:652. Originally published 2022 Jun 14. [Version 2] doi: 10.12688/f1000research.122328.2 (PMC9428500; doi:10.12688/f1000research.122328.2)
Supplement: Peer review history

# Peer review: Reviewer response for version 2

**DOI:** 10.5256/f1000research.137467.r148129

**Published:** 2022-08-30

**Authors:** Yuanyuan Zhou

**Affiliations:** 1 Evaluation, Assessment & Educational Research, Baylor College of Medicine, Houston, TX, USA

The second version has fully addressed my concerns.

Are sufficient details of methods and materials provided to allow replication by others?

Yes

Is the rationale for creating the dataset(s) clearly described?

Yes

Are the datasets clearly presented in a useable and accessible format?

Yes

Are the protocols appropriate and is the work technically sound?

Yes

Reviewer Expertise:

I am an experienced evaluation and assessment data analyst and medical school faculty member with a demonstrated history of accomplishment and growing responsibility in the Higher Education and Medical Education fields. I have devoted my time to educational program evaluation, performance assessment, curriculum review, continuous quality improvement, accreditation (both SACOCS and LCME), and educational research. I have intensive training and experience in quantitative and qualitative data analyses, which I utilize in my commitment to data-informed continuous improvement of curricula and learner-centered education. My primary research interest is in applying and expanding quantitative analysis methodologies for use in improving medical education.

I confirm that I have read this submission and believe that I have an appropriate level of expertise to confirm that it is of an acceptable scientific standard.

# Peer review: Reviewer response for version 2

**DOI:** 10.5256/f1000research.137467.r148130

**Published:** 2022-08-24

**Authors:** David Doncel-Abad

**Affiliations:** 1 Sociology and Communication, University of Salamanca, Salamanca, Spain

The authors have taken in account the suggestions made before.

Are sufficient details of methods and materials provided to allow replication by others?

Partly

Is the rationale for creating the dataset(s) clearly described?

Yes

Are the datasets clearly presented in a useable and accessible format?

Yes

Are the protocols appropriate and is the work technically sound?

Partly

Reviewer Expertise:

Sociology of Education and the analysis of public policy.

I confirm that I have read this submission and believe that I have an appropriate level of expertise to confirm that it is of an acceptable scientific standard.

# Peer review: Reviewer response for version 1

**DOI:** 10.5256/f1000research.134304.r143785

**Published:** 2022-08-12

**Authors:** David Doncel-Abad

**Affiliations:** 1 Sociology and Communication, University of Salamanca, Salamanca, Spain

**Rationale Dataset**

 The arguments for creating the database are fully explained. From the point of view of public policies, accountability is a relevant aspect both to evaluate a public policy and the performance of the Government. This argument justifies the development of a database for the evaluation of NYSC. However, in the specific case of the NYSC, it would be interesting to include whether there is a public debate on the purpose of this policy because it would further justify the suitability of creating the database to evaluate this politic.

**Protocols**

 The protocols followed for creating the sample are not optimal, but it is adequate because they have obtained a large sample. The snowball technique is an appropriate procedure to access members of the population that are difficult to contact because it is an economic resource. But among its drawbacks, we find the lack of control over how the sample is constituted. In addition, it is a technique that is particularly sensitive to sampling bias, for instance, to get enough representativeness of the population.

 In this case, the sample is large, which reduces the representativeness bias. However, it is highly recommended that the authors indicate the number of participants in the NYSC among the years 2012-2021 to demonstrate that the sample is large enough. In this way, a percentage of responses to the questionnaire could be calculated.

 Likewise, it is highly recommended that the authors explain how they have monitored an individual to not respond twice to the questionnaire when the questionnaire has been published on WhatsApp, Telegram, and Facebook. For these reasons, it is recommended that the article includes a reflection on why he has used snowball sampling and how the responses have been controlled.

 Regarding the variables, there is one in which I have a doubt, specifically, with the age cohorts: Can a Nigerian citizen be part of the NYSC program at the age of 16?

**Methods and Materials**

 There is enough detail in the article on how other researchers can apply the data. In addition, the authors provide the questions of the questionnaire and some proposals for lines of research.

**Format**

 The datasets are presented in a usable and accessible format. In fact, the authors make the database available in Excel, tabulated by commas, which allows the database to be used with different statistical packages such as SPSS, R, or STATA.

Are sufficient details of methods and materials provided to allow replication by others?

Partly

Is the rationale for creating the dataset(s) clearly described?

Yes

Are the datasets clearly presented in a useable and accessible format?

Yes

Are the protocols appropriate and is the work technically sound?

Partly

Reviewer Expertise:

Sociology of Education and the analysis of public policy.

I confirm that I have read this submission and believe that I have an appropriate level of expertise to confirm that it is of an acceptable scientific standard, however I have significant reservations, as outlined above.

# Response

**Published:** 2022-08-13

**Authors:** Valentine Owan

**Affiliations:** University of Calabar, Nigeria

**Comment #1:** The arguments for creating the database are fully explained. From the point of view of public policies, accountability is a relevant aspect both to evaluate a public policy and the performance of the Government. This argument justifies the development of a database for the evaluation of NYSC. However, in the specific case of the NYSC, it would be interesting to include whether there is a public debate on the purpose of this policy because it would further justify the suitability of creating the database to evaluate this politic.

**Response #1: **We are grateful to the reviewer for such a thoughtful suggestion. We have now provided the public debates and perceptions regarding the continuation/discontinuation of the NYSC programme in the introductory section.

**Comment #2:** The protocols followed for creating the sample are not optimal, but it is adequate because they have obtained a large sample. The snowball technique is an appropriate procedure to access members of the population that are difficult to contact because it is an economic resource. But among its drawbacks, we find the lack of control over how the sample is constituted. In addition, it is a technique that is particularly sensitive to sampling bias, for instance, to get enough representativeness of the population. In this case, the sample is large, which reduces the representativeness bias. However, it is highly recommended that the authors indicate the number of participants in the NYSC among the years 2012-2021 to demonstrate that the sample is large enough. In this way, a percentage of responses to the questionnaire could be calculated.

**Response #2:** Thank you for suggesting that we provide the NYSC annual intake. We agree that this information would have been useful in determining the degree of representative of the sample in our study for each year. However, just as we stated, the NYSC are very secretive with their data; one reason why the programme is rarely evaluated. What we do know is that NYSC camps in Nigeria can accommodate between 1000 to 4,000 corps members per batch, often three batches in a year and there are 37 camps nationwide. This range should give an idea about the number of graduates serving each year. Considering the large number of corps members that serve each year, this study does not make claims of having obtained a representative sample for each year. We do believe, however, that the respondents reached overall in this study is large enough to provide reliable information to assess the programme.

**Comment #3:** Likewise, it is highly recommended that the authors explain how they have monitored an individual to not respond twice to the questionnaire when the questionnaire has been published on WhatsApp, Telegram, and Facebook. For these reasons, it is recommended that the article includes a reflection on why he has used snowball sampling and how the responses have been controlled.

**Response #3:** We are very thankful to the reviewer for drawing our attention to this important aspect of ensuring data integrity. Be informed that in the revised version of the article. We have provided an explanation regarding how we assessed for malicious and multiple responses.

**Comment #4: **Regarding the variables, there is one in which I have a doubt, specifically, with the age cohorts: Can a Nigerian citizen be part of the NYSC program at the age of 16?

**Response #4:** We formed the age categories based on the minimum university entry age. To be admitted into any tertiary institution in Nigeria, a person must be sixteen years or older. At graduation, the youngest person should be 19 years depending on whether the programme duration was three or four years. Basically, individuals ticking that age category are considered to be between 19 and 20 years.

**Comment #5:** There is enough detail in the article on how other researchers can apply the data. In addition, the authors provide the questions of the questionnaire and some proposals for lines of research.

**Response #5:** We are grateful for your commendable review.

**Comment #6:** The datasets are presented in a usable and accessible format. In fact, the authors make the database available in Excel, tabulated by commas, which allows the database to be used with different statistical packages such as SPSS, R, or STATA.

**Response #6: **Thank you for reading our article and commenting on its strengths and weaknesses.

# Peer review: Reviewer response for version 1

**DOI:** 10.5256/f1000research.134304.r143786

**Published:** 2022-08-04

**Authors:** Yuanyuan Zhou

**Affiliations:** 1 Evaluation, Assessment & Educational Research, Baylor College of Medicine, Houston, TX, USA

I can see the value of the data set and how publishing it could potentially benefit other scholars for similar research topics. There are two places that might need the authors to address.

1. Under Methods - Ethics: "In addition, all biodata, including age, education, and experience, were represented as a range of equal intervals."
  It is not clear how biodata can be treated as equal intervals. I checked the data file, "AGE" was coded as "1", "2", "3", and "4", "GENDER" was coded as "1" and "2", "Education" was coded as "1", "2", "3", and "4, "but no additional explanation was provided. And even though there are additional labels for each level of data, I would argue that some variables are more like ordinal scaled variables.
2. With such a long survey (52 questions) and such a significant among of respondents, it is rare to see no missing among the data set. If the respondents were required to answer all questions, it should be better to mention it under the data collection section.

Are sufficient details of methods and materials provided to allow replication by others?

Yes

Is the rationale for creating the dataset(s) clearly described?

Yes

Are the datasets clearly presented in a useable and accessible format?

Yes

Are the protocols appropriate and is the work technically sound?

Yes

Reviewer Expertise:

I am an experienced evaluation and assessment data analyst and medical school faculty member with a demonstrated history of accomplishment and growing responsibility in the Higher Education and Medical Education fields. I have devoted my time to educational program evaluation, performance assessment, curriculum review, continuous quality improvement, accreditation (both SACOCS and LCME), and educational research. I have intensive training and experience in quantitative and qualitative data analyses, which I utilize in my commitment to data-informed continuous improvement of curricula and learner-centered education. My primary research interest is in applying and expanding quantitative analysis methodologies for use in improving medical education.

I confirm that I have read this submission and believe that I have an appropriate level of expertise to confirm that it is of an acceptable scientific standard, however I have significant reservations, as outlined above.

# Response

**Published:** 2022-08-13

**Authors:** Valentine Owan

**Affiliations:** University of Calabar, Nigeria

**Comment #1: **Under Methods - Ethics: "In addition, all biodata, including age, education, and experience, were represented as a range of equal intervals." It is not clear how biodata can be treated as equal intervals. I checked the data file, "AGE" was coded as "1", "2", "3", and "4", "GENDER" was coded as "1" and "2", "Education" was coded as "1", "2", "3", and "4, "but no additional explanation was provided. And even though there are additional labels for each level of data, I would argue that some variables are more like ordinal scaled variables.

**Response #1: **We are grateful to the reviewer for vetting our manuscript. We have reviewed the sentence and have revised it to convey our idea more clearer. We agree also with the reviewer that some variables are nominal, ordinal and scaled. Age, for example, can take discrete, ordinal, or continuous forms depending on how it was conceptualised and measured. In this study, we treated age ordinally by creating different ranges of groups for respondents to tick where their age bracket falls. If we wanted, we could have asked respondents to provide their age in years. Gender is a nominal variable with two levels. Education level is ordinal with four levels. We did not provide these details because the journal’s guidelines for data notes do not permit the interpretation of tables. Furthermore, just as you noted, the dataset already contains these.

**Comment #2:** With such a long survey (52 questions) and such a significant among of respondents, it is rare to see no missing among the data set. If the respondents were required to answer all questions, it should be better to mention it under the data collection section.

**Response #2: **We stated that there was no missing data because all the questions were compulsory except for the optional follow-up question. This was stated in the data collection section.
